# Understanding How Exercise Promotes Cognitive Integrity in the Aging Brain

**DOI:** 10.1371/journal.pbio.1002300

**Published:** 2015-11-11

**Authors:** Benjamin M. Laitman, Gareth R. John

**Affiliations:** Friedman Brain Institute, Icahn School of Medicine at Mount Sinai, New York, New York, United States of America

## Abstract

Alterations in the structure and organization of the aging central nervous system (CNS), and associated functional deficits, result in cognitive decline and increase susceptibility to neurodegeneration. Age-related changes to the neurovascular unit (NVU), and their consequences for cerebrovascular function, are implicated as driving cognitive impairment during aging as well as in neurodegenerative disease. The molecular events underlying these effects are incompletely characterized. Similarly, the mechanisms underlying effects of factors that reduce the impact of aging on the brain, such as physical exercise, are also opaque. A study in this issue of *PLOS Biology* links the NVU to cognitive decline in the aging brain and suggests a potential underlying molecular mechanism. Notably, the study further links the protective effects of chronic exercise on cognition to neurovascular integrity during aging.

## Abstract 2

This Primer examines recent evidence that enhances our understanding of how aging and exercise differentially affect brain function via their impact on the neurovascular unit.

## Introduction

Age-associated alterations in the structure and organization of the central nervous system (CNS) result in cognitive impairment [1] and increase susceptibility to individual overt neurodegenerative pathology and clinical disease [2]. Thus, understanding the factors that contribute to age-related changes in structure and function within the CNS may lead to novel approaches to prevent the associated cognitive decline as well as reduce the incidence of neurodegenerative conditions such as Alzheimer disease (AD).

## Links between Cerebrovascular Function and Cognitive Function in Aging

**Fig 1:**
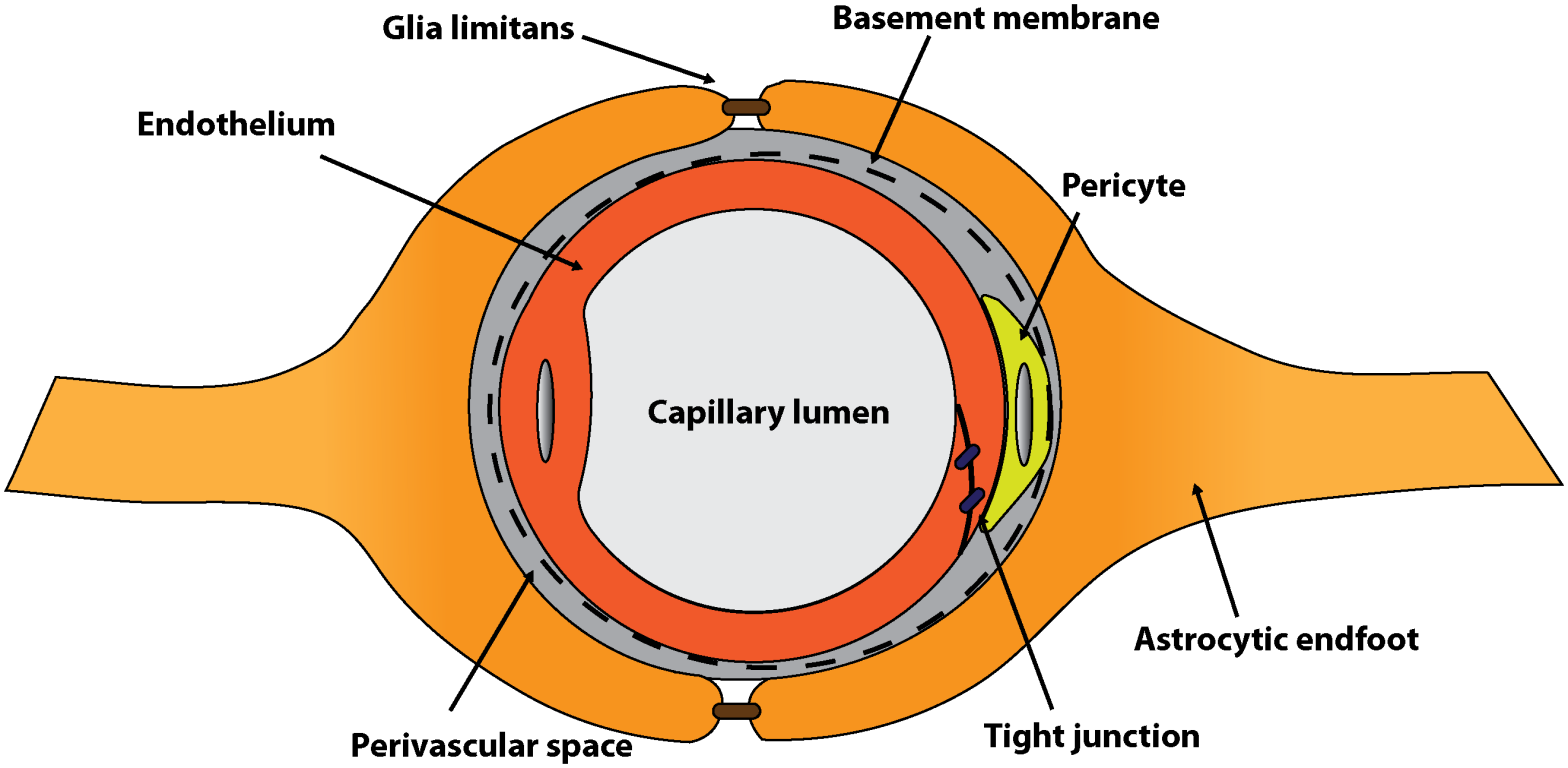
Schematic of the NVU. The NVU comprises the cerebral microvascular endothelium (shown in red), its basement membrane, and associated pericytes (yellow) and astrocytes (orange). The perivascular space exists between the endothelium and astrocytic endfeet. The endothelium provides the structural and functional basis for the blood–brain barrier (BBB), while astrocytes and pericytes control barrier induction and maintenance [7]. Junctional proteins exist between endothelial cells and astrocytes (glia limitans) to help regulate entrance into the CNS parenchyma. *Image credit*: *Gareth R*. *John & Benjamin M*. *Laitman*.

Changes in both neuronal and non-neuronal populations within the CNS have been associated with functional deficits in the aging brain. For example, alterations in synaptic morphology and activity of neurons, notably in the hippocampus and prefrontal cortex, have been associated with impairments in cognition during aging [3]. Notably, and perhaps less intuitively, important roles for non-neuronal lineages have also been demonstrated in cognitive decline. Chronic inflammatory changes in resident and invading cells of the innate immune system, such as microglia/monocytes and astrocytes, occur with aging [4] and may predispose to neural pathology in the CNS. In addition, age-related changes to the neurovascular unit (NVU), (Fig 1) and associated cerebrovascular alterations, have also been implicated as impacting neural integrity and function [5,6]. However, the relative contribution of alterations of the NVU to these age-related changes in brain structure and function, and the molecular events that drive them, are incompletely characterized. Thus, an important question is how exactly cerebrovascular function, particularly integrity of the NVU, impacts cognitive function during aging. Examination of underlying mechanisms presents an interesting avenue to discover and develop treatments to prevent cognitive decline and/or neurodegeneration.

The NVU comprises the cerebral microvascular endothelium, its basement membrane, and associated pericytes and astrocytes [7,8] (Fig 1). The endothelium provides the structural and functional basis for the blood–brain barrier (BBB), while astrocytes and pericytes control barrier induction and maintenance [7,8]. Notably, defects in the NVU have been linked to cognitive decline in the context of neurodegenerative conditions. For example, vascular defects in mice deficient for the AD risk factor, apolipoprotein E (ApoE), or transgenic for the human *APOE4* isoform, display vascular defects that precede neuronal dysfunction [9]. Furthermore, human *APOE4* carriers may be susceptible to age-dependent breakdown of the BBB prior to the onset of clinical AD deterioration [10].

Studies have also linked neurovascular dysfunction to cognitive decline in aging. Cerebral microvascular pathology together with reductions in cerebral blood flow (CBF) and glucose and oxygen metabolism are known to occur during human aging [11]. Moreover, use of a novel high-resolution MRI technique has also shown that vascular leakage is an early event in the aged human brain, beginning in the hippocampus and correlating with cognitive deficit [12]. These studies, and others like them, suggest that changes in the NVU may be significant in driving deterioration in the structure and organization of the CNS, and therefore cognitive deficits, during aging as well as in neurodegenerative disease.

## The Mechanism by Which Exercise Impacts the Aging Brain Is Incompletely Understood

**Fig 2:**
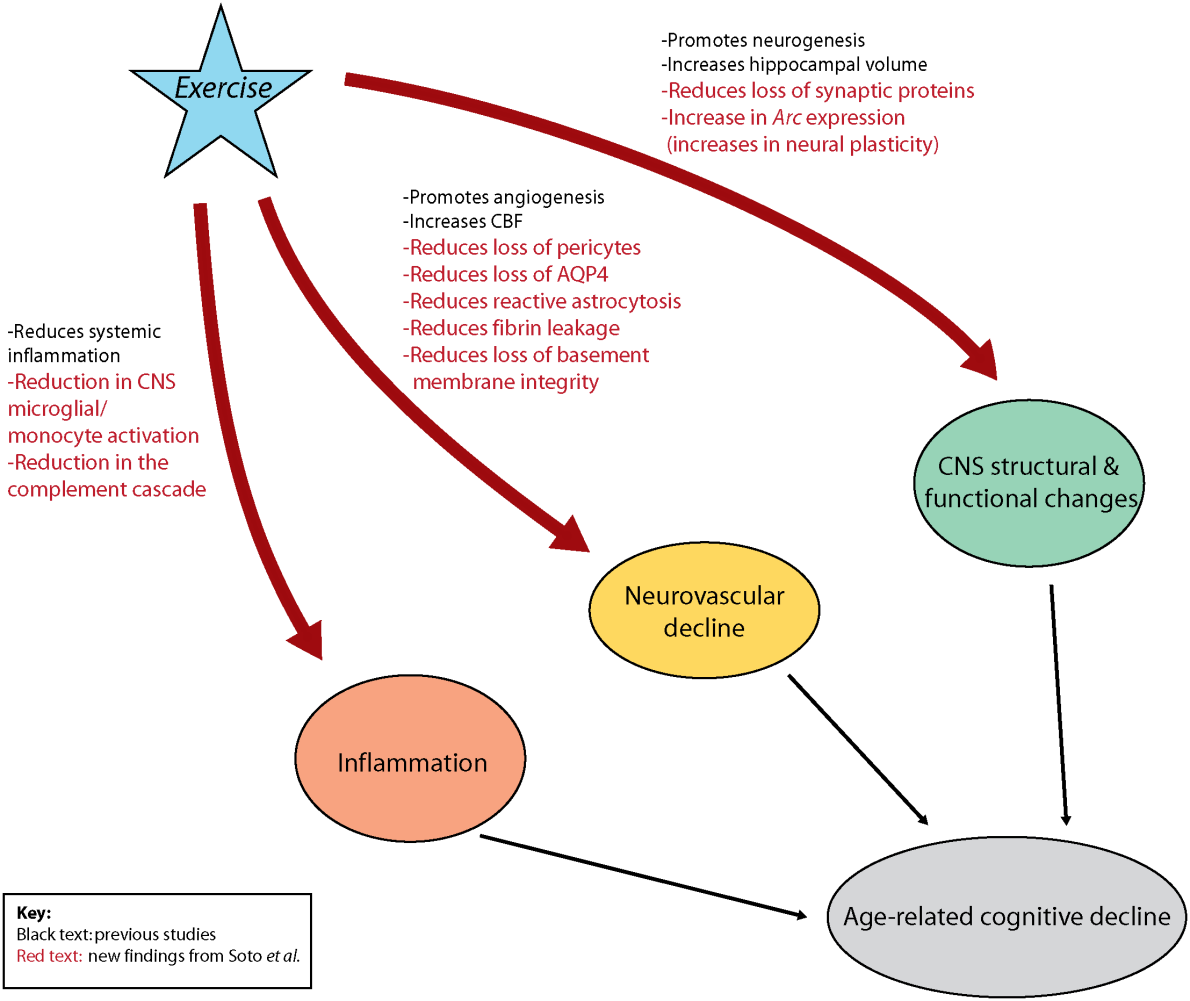
The impacts of exercise on age-related cognitive decline. Three major factors have been implicated in promoting age-related cognitive decline: inflammation, neurovascular changes, and changes in CNS structure and function. Exercise has been shown to be beneficial in impacting these three categories. It has been shown to promote neurogenesis [16], increase CBF and angiogenesis [14], and reduce inflammation [4,17], correlating with improved cognitive performance. Soto et al. adds to this data (red text) by demonstrating that exercise reduces the age-related loss of pericytes, basement membrane components, and astrocyte reactivity at the NVU, and reduces the amount of complement induction in myeloid cells. *Image credit*: *Gareth R*. *John & Benjamin M*. *Laitman*.

Interestingly, the mechanisms underlying effects of factors that reduce the impact of aging on the brain are similarly opaque. A good example is physical exercise, which is strongly associated with protection against age-related decline in cognitive and sensorimotor function [1,13] (Fig 2). Exercise is known to improve cardiovascular function, and blood flow within the CNS, and is associated with improved cognitive, sensory, and motor test outcomes, angiogenesis, and neurogenesis [13–16]. However, the molecular events that drive this protective effect remain incompletely understood.

A study in this issue of *PLOS Biology* by Soto and coauthors builds on these previous findings, linking the NVU to cognitive decline in the aging brain and suggesting a potential underlying molecular mechanism [18]. Notably, the study further links the protective effects of exercise on cognition to neurovascular integrity. The authors present RNA sequencing data from young (4 mo) and aged (21 mo) mice that suggest that aging causes regional compromise of the NVU. They confirm these findings using immunohistochemistry and electron microscopy, which reveals deterioration of neurovascular structures in the aging brain, including basement membrane reduction, loss of pericytes, and astrocyte dysfunction. Importantly, these neurovascular changes result in vascular leakage. Interestingly, these events further correlate with microglial/monocyte activation and complement induction, implicated in age-related cognitive decline in mice [19]. Collectively, these data suggest a connection between neurovascular dysfunction and innate inflammation in the context of cognitive decline in aging. The authors then turn their focus to the long-term impact of aerobic exercise on age-related neurovascular dysfunction. They show that exercise strongly protects against neurovascular decline in aged mice, and that this correlates with improved behavioral outcomes and markers of neuroplasticity and reduced inflammation and complement induction (Fig 2).

What are the molecular mechanisms responsible for these outcomes? Interestingly, the authors’ data in the final section of their paper returns us to ground familiar to those in the fields of neurodegeneration and AD. Their findings implicate the AD risk factor ApoE as also regulating neurovascular integrity during aging and, moreover, suggest involvement of ApoE in the protective effects of exercise. They show that astrocytic ApoE decreases in aged mice concomitant with age-related neurovascular decline and complement activation, and that this effect is prevented by exercise. These data extend previous studies showing that ApoE deficiency in mice leads to progressive age-related neurovascular dysfunction [9,20,21] and suggest a potential link between astrocytic ApoE, age-related neurovascular dysfunction, and microglial/monocyte activation. To test this hypothesis, the authors examine the effect of regular exercise from life to old age on ApoE-deficient mice. Importantly, In contrast to wild-type mice, they show that exercise has no effect on age-related neurovascular decline or microglia/monocyte activation in the absence of ApoE.

## Future Directions

Collectively, the findings presented by Soto et al. connect the NVU to cognitive decline in the aging brain and suggest that exercise can prevent or attenuate age-related indices of both neurovascular decline and inflammation. They further propose that the beneficial effects of exercise on neurovascular integrity and inflammation, and neuroplasticity, may be mediated at least in part by ApoE. How then might these findings be extended, and what might be the logical next steps in the current work? Several interesting questions spring to mind, some of which are discussed by the authors. For example, do the increases in microglia/monocytes seen in aging mice in the current study occur as a result of proliferation of resident microglia, infiltration of peripheral monocytes, or both? How do these responses impact cognitive function during aging, given that increase of proinflammatory markers in the blood is correlated with poor cognitive performance in older adults? And does complement induction seen in aged mice precede neurovascular decline, or occur as a consequence to it?

An additional point that should be addressed is how this work extends to age-related cognitive decline in humans. While several studies, including Soto et al., provide encouraging evidence of the positive effects of exercise on age-related cognitive decline, findings in humans have been inconsistent. In a study that examined a combination of dietary changes and aerobic exercise, the authors found exercise only improved cognition when combined with a high flavanol diet [22]. Unexpectedly, the exercise program alone failed to improve cognition. While this same research group has previously demonstrated exercise to be beneficial for human cognitive tasks [16], the authors bring up the possibility that the exercise regimen used in their diet-exercise study was not stringent enough for the older population investigated. The benefit of exercise on age-related cognitive decline is still inconsistent in humans, even if it strongly protects against the cognitive decline in aged rodents. More robust studies in human populations are thus warranted.

However, perhaps the most important questions that the current study suggests are those that are more directly translational. Notably, what is the detailed molecular mechanism of action by which ApoE preserves neurovascular integrity and cognitive function in the aging brain, and can we develop targeted therapies based on those results? Based on recent studies, might neurovascular and cognitive decline in the brain be reduced by pharmacologically targeting the cyclophilin A-nuclear factor κB-matrix metalloproteinase-9 axis, as has been proposed for *APOE4*-mediated neurovascular injury [9]? Furthermore, as noted by the authors, the constitutive ApoE-deficient mice used in the manuscript already displayed a dysfunctional NVU prior to the start of exercise studies, in contrast to wild-type mice. Thus, it would be interesting to repeat these studies using an inducible conditional allele. This may answer the question of whether, in addition to protecting against neurovascular dysfunction in aging mice, exercise might be used as an intervention to repair (or restore) existing neurovascular breakdown. Such findings could be translationally useful, in the prevention or amelioration of age-related and pathologic cognitive decline in the growing geriatric population.

## Abbreviations

AD: Alzheimer disease
ApoE: apolipoprotein E
BBB: blood–brain barrier
CBF: cerebral blood flow
CNS: central nervous system
NVU: neurovascular unit

## References

[1] Seidler R.D., et al, Motor control and aging: links to age-related brain structural, functional, and biochemical effects. Neurosci Biobehav Rev, 2010 34(5): p. 721–33. 10.1016/j.neubiorev.2009.10.005 19850077 PMC2838968

[2] Yankner B.A., Lu T., and Loerch P., The aging brain. Annu Rev Pathol, 2008 3: p. 41–66. 18039130 10.1146/annurev.pathmechdis.2.010506.092044

[3] Morrison J.H. and Baxter M.G., The ageing cortical synapse: hallmarks and implications for cognitive decline. Nat Rev Neurosci, 2012 13(4): p. 240–50. 10.1038/nrn3200 22395804 PMC3592200

[4] Ownby R.L., Neuroinflammation and cognitive aging. Curr Psychiatry Rep, 2010 12(1): p. 39–45. 10.1007/s11920-009-0082-1 20425309

[5] Zlokovic B.V., Neurodegeneration and the neurovascular unit. Nat Med, 2010 16(12): p. 1370–1. 10.1038/nm1210-1370 21135839

[6] de la Torre J.C., Vascular risk factors: a ticking time bomb to Alzheimer's disease. Am J Alzheimers Dis Other Demen, 2013 28(6): p. 551–9. 10.1177/1533317513494457 23813612 PMC10852736

[7] Abbott N.J., Ronnback L., and Hansson E., Astrocyte-endothelial interactions at the blood-brain barrier. Nat Rev Neurosci, 2006 7(1): p. 41–53. 16371949 10.1038/nrn1824

[8] Lo E.H. and Rosenberg G.A., The neurovascular unit in health and disease: introduction. Stroke, 2009 40(3 Suppl): p. S2–3. 10.1161/STROKEAHA.108.534404 19064779 PMC2811575

[9] Bell R.D., et al, Apolipoprotein E controls cerebrovascular integrity via cyclophilin A. Nature, 2012 485(7399): p. 512–6. 10.1038/nature11087 22622580 PMC4047116

[10] Halliday M.R., et al, Relationship between cyclophilin a levels and matrix metalloproteinase 9 activity in cerebrospinal fluid of cognitively normal apolipoprotein e4 carriers and blood-brain barrier breakdown. JAMA Neurol, 2013 70(9): p. 1198–200. 10.1001/jamaneurol.2013.3841 24030206 PMC4047029

[11] Farkas E. and Luiten P.G., Cerebral microvascular pathology in aging and Alzheimer's disease. Prog Neurobiol, 2001 64(6): p. 575–611. 11311463 10.1016/s0301-0082(00)00068-x

[12] Montagne A., et al, Blood-brain barrier breakdown in the aging human hippocampus. Neuron, 2015 85(2): p. 296–302. 10.1016/j.neuron.2014.12.032 25611508 PMC4350773

[13] Mattson M.P., Energy intake and exercise as determinants of brain health and vulnerability to injury and disease. Cell Metab, 2012 16(6): p. 706–22. 10.1016/j.cmet.2012.08.012 23168220 PMC3518570

[14] Ding Y.H., et al, Cerebral angiogenesis and expression of angiogenic factors in aging rats after exercise. Curr Neurovasc Res, 2006 3(1): p. 15–23. 16472122 10.2174/156720206775541787

[15] Erickson K.I., et al, Exercise training increases size of hippocampus and improves memory. Proc Natl Acad Sci U S A, 2011 108(7): p. 3017–22. 10.1073/pnas.1015950108 21282661 PMC3041121

[16] Pereira A.C., et al, An in vivo correlate of exercise-induced neurogenesis in the adult dentate gyrus. Proc Natl Acad Sci U S A, 2007 104(13): p. 5638–43. 17374720 10.1073/pnas.0611721104 PMC1838482

[17] Cotman C.W., Berchtold N.C., and Christie L.A., Exercise builds brain health: key roles of growth factor cascades and inflammation. Trends Neurosci, 2007 30(9): p. 464–72. 17765329 10.1016/j.tins.2007.06.011

[18] Soto I., et al, APOE stabilization by exercise prevents aging neurovascular dysfunction and complement induction. PLoS Biol, 2015. 10.1371/journal.pbio.1002279 PMC4626092 26512759

[19] Stephan A.H., et al, A dramatic increase of C1q protein in the CNS during normal aging. J Neurosci, 2013 33(33): p. 13460–74. 10.1523/JNEUROSCI.1333-13.2013 23946404 PMC3742932

[20] Raber J., et al, Isoform-specific effects of human apolipoprotein E on brain function revealed in ApoE knockout mice: increased susceptibility of females. Proc Natl Acad Sci U S A, 1998 95(18): p. 10914–9. 9724804 10.1073/pnas.95.18.10914 PMC27995

[21] Buttini M., et al, Expression of human apolipoprotein E3 or E4 in the brains of Apoe-/- mice: isoform-specific effects on neurodegeneration. J Neurosci, 1999 19(12): p. 4867–80. 10366621 10.1523/JNEUROSCI.19-12-04867.1999 PMC6782676

[22] Brickman A.M., et al, Enhancing dentate gyrus function with dietary flavanols improves cognition in older adults. Nat Neurosci, 2014 17(12): p. 1798–803. 10.1038/nn.3850 25344629 PMC4940121

